# Accuracy of a Dual-Sensor Heat-Flux (DHF) Non-Invasive Core Temperature Sensor in Pediatric Patients Undergoing Surgery

**DOI:** 10.3390/jcm12227018

**Published:** 2023-11-09

**Authors:** Sebastian Zeiner, Markus Zadrazil, Harald Willschke, Marion Wiegele, Peter Marhofer, Fabian Peter Hammerle, Daniel Laxar, Andreas Gleiss, Oliver Kimberger

**Affiliations:** 1 Department of Anaesthesia, Intensive Care Medicine and Pain Medicine, Medical University of Vienna, 1090 Vienna, Austriamarkus.zadrazil@meduniwien.ac.at (M.Z.); peter.marhofer@meduniwien.ac.at (P.M.); oliver.kimberger@meduniwien.ac.at (O.K.); 2 Ludwig Boltzmann Institute Digital Health and Patient Safety (LBI DHPS), 1090 Vienna, Austria; 3 Institute of Clinical Biometrics, Center for Medical Data Science, Medical University of Vienna, 1090 Vienna, Austria

**Keywords:** pediatric, anesthesia, temperature management, hypothermia, temperature measurement, perioperative care

## Abstract

Accurate temperature measurement is crucial for the perioperative management of pediatric patients, and non-invasive thermometry is necessary when invasive methods are infeasible. A prospective observational study was conducted on 57 patients undergoing elective surgery. Temperatures were measured using a dual-sensor heat-flux (DHF) thermometer (Tcore™) and a rectal temperature probe (TRec), and the agreement between the two measurements was assessed. The DHF measurements showed a bias of +0.413 °C compared with those of the TRec. The limits of agreement were broader than the pre-defined ±0.5 °C range (−0.741 °C and +1.567 °C). Although the DHF sensors tended to overestimate the core temperature compared to the rectal measurements, an error grid analysis demonstrated that 95.81% of the DHF measurements would not have led to a wrong clinical decision, e.g., warming or cooling when not necessary. In conclusion, the low number of measurements that would have led to incorrect decisions suggests that the DHF sensor can be considered an option for continuous temperature measurement when more invasive methods are infeasible.

## 1. Introduction

Body temperature is important for maintaining normal physiological functions. Inadvertent hypothermia, as well as pyrexia, is associated with higher morbidity and mortality [1,2,3]. Consequently, continuous measurement of the body is an integral part of standard monitoring in the context of perioperative patient care [4,5].

Neonates and infants are particularly vulnerable to thermal variations. In comparison to adults, children lose a significant percentage of their core heat through non-evaporative heat loss mechanisms such as conduction, convection and radiation [6]. As a result, children are at a higher risk of hypothermia. Perioperative hypothermia is common and occurs in around 45% of infants undergoing surgery [7,8]. Hypothermia in pediatric patients can result in a range of adverse effects, from discomfort to increased morbidity and mortality [7,9,10]. While data are limited, hypothermic pediatric patients are likely impacted by factors such as medication responses, clotting, cardiac functions, wound healing and thermal discomfort [11,12,13].

Although data are lacking, it is reasonable to assume that children experience similar negative effects of hypothermia to those of adults. On the other hand, the utilization of aggressive forced-air warming techniques has introduced a novel concern known as iatrogenic intraoperative hyperthermia in the pediatric population [14]. Intraoperative hyperthermia is characterized by a body core temperature surpassing 38.0 °C [15,16]. This condition can result in heightened basal metabolic rates and sympathetic activity, leading to elevated oxygen requirements [17], and is associated with an escalated susceptibility to surgical site infections among infants [10].

The use of core temperature measurements such as pulmonary catheters (gold standard) and esophageal, nasopharyngeal or rectal probes is recommended because of their higher accuracy than that of non-invasive measuring methods, and these methods are considered the standard of care [7,18,19]. Skin temperature measurements are often used to estimate core temperature for practical reasons but are biased toward lower-than-core temperatures and can only poorly detect rapid core temperature changes [20,21].

Contrary to temperature management in adults, the use of rectal probes for temperature measurement is recommended for neonates and small children, as stated in the German, Austrian and Swiss guidelines for perioperative temperature management [4]. It is one of the recommended approaches for detecting hypothermia during the pre-, intra- and perioperative phases in small children [7,19].

Non-invasive core thermometers aim to combine the advantages of skin and core measurements. Three non-invasive core thermometers are currently available for clinical use, each utilizing a slightly different technology: a zero-heat-flux (ZHF) thermometer (3M™ Bair Hugger™ temperature monitoring system, formerly 3M SpotOn system; 3M, Saint Paul, MN, USA) and two dual-sensor heat-flux (DHF) thermometers, the Tcore™ (Dräger, Drägerwerk AG & Co. KG, Lübeck, Germany) and Temple Touch Pro™ (Medisim Ltd., Beit-Shemesh, Israel). ZHF thermometers were first developed almost 50 years ago [22]. These thermometers work via simulating absolute insulation on the body surface, leading to an elimination of evaporation and convection and establishing an isothermal tunnel from the body’s core to the surface, thus allowing core temperature measurement on the skin [20,23,24,25,26,27]. In comparison, DHF thermometers consist of two sensors separated by a known thermal resistance. One side is placed directly on the patient’s skin and the other faces the environment. After some equilibration time, the core temperature can thus be calculated [26,28].

The Temple Touch Pro™ and the 3M™ Bair Hugger™ temperature monitoring systems have been tested for pediatric patients [7,29,30,31]; however, to date, no study exists of the use of Tcore thermometer technology in neonates and infants.

Therefore, the objective of this study was to evaluate the accuracy of the DHF sensor in measuring core temperatures in the pediatric population during the intraoperative period. We assessed the levels of agreement between DHF temperature measurements and rectal temperatures in children of up to 6 years of age.

## 2. Materials and Methods

### 2.1. Design

This study was designed as an investigator-initiated, single-center prospective observational study of infants, toddlers and small children (<6 years of age) undergoing elective surgery, comparing measurements from the Tcore™ thermometer with measurements from a rectal temperature probe, which is the standard of care in our center.

### 2.2. Ethics and Registration

This study received approval from the Institutional Ethics Committee of the Medical University of Vienna (protocol code 2162/2021 on 27 April 2022). Informed consent was obtained from the legal guardians of all participating patients. This study was conducted in accordance with the Declaration of Helsinki and the Good Clinical Practice guidelines. The trial was registered with the Austrian Federal Office for Safety in Health Care (reference no.: 100947856, registered on 1 June 2022) in compliance with Austrian and European Law. Moreover, this study was registered on ClinicalTrials.gov with the identifier NCT05516771.

### 2.3. Patients

All patients under the age of 6 years who underwent surgeries, at the Department of Pediatric and Adolescent Surgery of the Medical University of Vienna between 18 October 2022 and 23 February 2023, requiring the insertion of a rectal temperature probe as a routine method of temperature measurement were eligible for inclusion. Patients and their legal guardians were invited to participate, and informed consent was obtained. The participants had the freedom to withdraw from this study at any time without this affecting their subsequent treatment. Exclusion criteria included rashes, infections and procedures that would have impeded the application of the heat flux thermometers; a fragile forehead skin state; a known allergy to the probe adhesive or any constituent components, maxillofacial trauma or lesions; neurological impairments with abnormal thermoregulation; hemodynamic instability; the need for vasoactive medication; procedures associated with extended use of abdominal/thoracic rinsing fluids; malignant hyperthermia or a family history of malignant hyperthermia; fevers and infections; and all conditions that might be judged to abnormally alter skin perfusion.

A pediatric convective underbody blanket (3M, Saint Paul, MN, USA) was used for all patients. The room temperature was set to 26 °C, as is the standard of care at the Department of Pediatric and Adolescent Surgery.

### 2.4. Measurements

Temperature measurements were obtained using a dual-sensor heat-flux (DHF) thermometer, the Tcore™ (T_DHF_) (Dräger, Drägerwerk AG & Co. KG, Lübeck, Germany), and via a rectally placed Medical Level 1 disposable General Purpose Temperature Probe ER400 (T_Rec_) (Smiths Medical Österreich GmbH, Brunn am Gebirge, Austria). The “Instructions for use” of the Tcore™ non-invasive core temperature sensor specify an accuracy between 25 °C and 45 °C, with ±0.1 °C [32]. The Medical Level 1 disposable General Purpose Temperature Probe ER400’s “Instructions for use” specify an accuracy between 5 °C and 45 °C, with ±0.2 °C [33]. Rectal temperature probes were placed according to routine practice: first lubricated, then placed 4–5 cm past the sphincter and into the rectum. The Tcore™ thermometer is an oval, with dimensions of 5 cm by 6 cm. This design allows it to comfortably fit on a toddler’s forehead. The sensors were positioned above the eye and the sinus frontalis. Both probes were placed immediately after the induction of anesthesia. The care-providing anesthesiologists and surgeons were not blinded to the measurements from the Tcore™ Thermometer. However, they were informed about the experimental nature of these measurements and instructed to guide the temperature management according to the measurements of the rectal probe and the department’s standard of care.

Temperature measurements were automatically recorded and transmitted to the IntelliSpace Critical Care and Anesthesia (ICCA; Philips GmbH Healthcare, Amsterdam, The Netherlands) database in 2 min intervals. After the data extraction, the first two measurements were discarded to enable equilibration. Changes in body temperature of more than 1 degree between two 2 min intervals and measurements below 30° were considered probe dislocation, and all subsequent measurements were discarded.

### 2.5. Statistical Methods

Statistical planning was performed by the Institute of Clinical Biometrics at the Center for Medical Data Science of the Medical University of Vienna. Upon study completion, datapoints were extracted from the IntelliSpace Critical Care and Anesthesia database into Excel (Microsoft Office Excel 2019; Microsoft, Redmond, WA, USA).

The primary outcome was the agreement analysis of the T_DHF_ sensor against the reference of T_Rec_ in 2 min intervals. Deviations of ±0.5 °C between T_DHF_ and T_Rec_ were considered acceptable, and a standard error of 0.05 °C was considered sufficient for the estimated limits of agreement.

In order to obtain an approximate estimate for a meaningful sample size, we made the following assumptions. If only one combination of measurements were recorded per patient and the aforementioned acceptable deviation and standard error hold true, this would result in 74 patients [34], if the between-subject standard deviation was assumed as 0.25 °C (half the acceptable deviation of 0.5 °C; lower standard deviations would result in higher precision for the limits of agreement). We estimated to observe a median of 20 measurements per patient, resulting in a lower sample size than with non-repeating measurements. However, since the variability between measurements for the same patient (and method) over time was unknown but could exhibit a considerable impact on the final sample size, we used the first 50 patients to estimate the within-patient variability.

Based on this information and the above-listed a priori settings (namely acceptable deviation and limits of agreement), we calculated the final sample size by inverting the equation of Zou et al. with data from the initial 51 patients [35], resulting in a final sample size of 117 patients, significantly higher than the prior anticipated amount of 74 patients budgeted by our department and approved by the Ethics Committee. Inclusion was therefore stopped early after the initial 51 patients.

### 2.6. Outcome Analysis

The agreements between the T_DHF_ and T_Rec_ measurements were evaluated through a Bland–Altman plot of T_DHF_ and standard measurement (T_Rec_). Limits of agreement were determined using the method of Zou [35]. Consistently with similar studies, clinically acceptable deviation was defined as 95% of difference within ±0.5 °C compared with the reference measurement [36].

Additionally, an error grid analysis was performed to determine whether measurement differences would result in incorrect clinical decisions [7,27]. The error grid zones were defined as follows:

Zone A was defined as an area of a ±0.5 °C error on either side of the T_Rec_ measurements. Measurement errors smaller than  ±0.5 °C were considered clinically not relevant [7,31,36]. Zone B was the area where measurement errors would exceed 0.5 °C but not lead to wrong clinical decisions; e.g., if T_Rec_ were 36.5 °C and T_DHF_ showed a temperature of 37.4 °C, neither temperature would lead to active warming therapy or a diagnostic workup for infection. In contrast, Zone C indicated errors that would lead to wrong clinical decisions and may harm a patient; e.g., if T_Rec_ were 36.5 °C and T_DHF_ showed 34.0 °C, the patient would receive active warming, although not indicated [31]. Region D was the points indicating a potentially dangerous failure to detect a need for treatment that could prevent hypothermia, defined as below 35.6 °C, or hyperthermia, defined as above 37.9 °C in T_Rec_ but not in T_DHF_ [27,31,37]. Since the standard of care in this population is an underbody blanket and a room temperature of 26 °C, we assumed only temperatures below 35.6 °C or above 37.9 °C would lead to additional treatment [15]. A recent multicenter study showed no outcome difference between patients at 35.5 °C and at 37 °C; however, severe hypothermia below 35 °C needs to be avoided, and countermeasures should be taken before a patient reaches such a low core temperature [38]. Zone E was all points that would lead to the opposite of the correct treatment: for example, a T_DHF_ of 35.3 °C and a T_Rec_ of 38.4°, meaning a patient that should have been cooled would receive additional warming, or vice versa [31].

Analyses were performed using SAS 9.4M6 (SAS Institute Inc., 2016, Cary, NC, USA). Error grid analysis was performed using python 3.8.5 with the pandas 1.3.4 package [39].

## 3. Results

**Figure 1:**
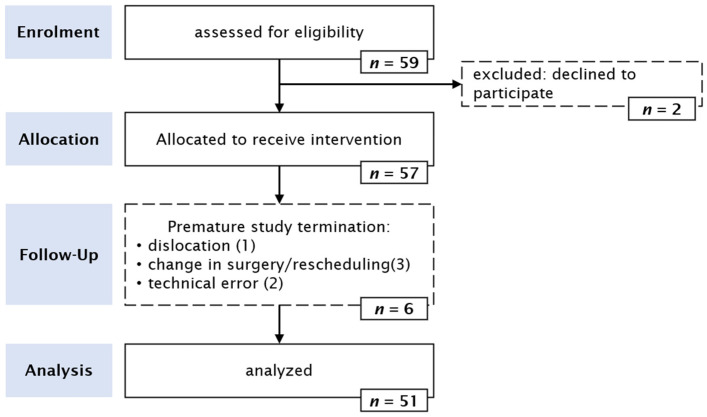
Patient flow chart.

Between October 2022 and February 2023, a total of 57 patients were enrolled in this study (see Figure 1). Six patients were subsequently excluded from this analysis; one patient experienced dislocation of the rectal probe after the initial three measurements, one patient was lost due to surgery rescheduling, two patients underwent changes in surgery that prevented accurate rectal measurements and two patients encountered technical difficulties resulting in temperature data being gathered only from the Tcore site. The remaining 51 patients contributed a median number of 26 measurements (quartiles: 16, 39).

The median patient age (quartiles) was 18.80 (9.37–44.73) months. Only five of the included patients were female and forty-six were male. The median patient weight was 11.00 (8.50–14.00) kgBW. Additional characteristics are displayed in Table 1. The final dataset consisted of 1646 data pairs.

The measurements of T_DHF_ ranged from 35.1 °C to 38.8 °C, with a median (1st–3rd quartile) of 37.1 °C (36.7–37.5 °C). The T_Rec_ measurements ranged from 34.4 °C to 37.9 °C, with a median of 36.7 (36.3–37.1 °C).

T_DHF_ had, on average, a temperature 0.413 °C higher than T_Rec_. In addition, the limits of agreement, at ±1.154 °C around this bias, were substantially broader than the predefined ±0.5 °C. The standard error for the limits of agreement was 0.076 °C instead of the 0.05 °C targeted in the protocol.

**Figure 2:**
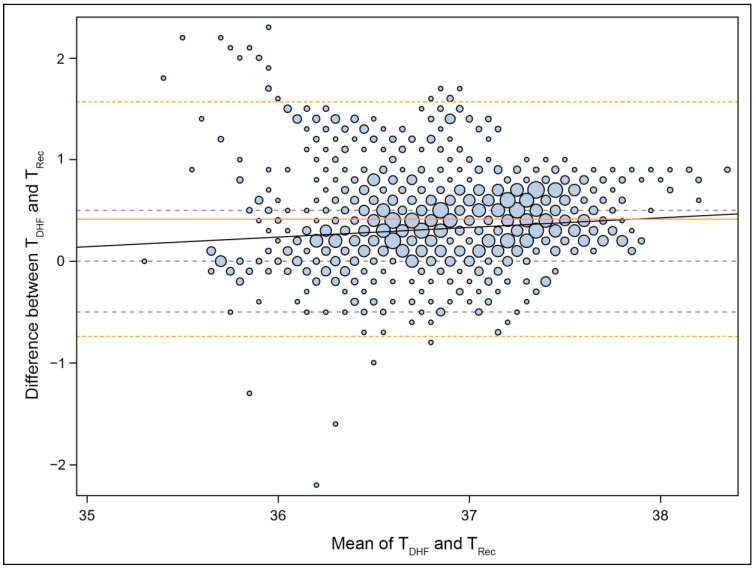
Bland–Altman Plot: This plot illustrates the agreement between the T_DHF_ and T_Rec_ measurements. The sizes of the data points correspond to the number of observations they represent. The central gray dashed line indicates 0 °C (no difference), while the additional gray dashed lines at ±0.5 °C represent predefined tolerated deviations. The blue line represents a linear trend, while the solid orange line represents the mean difference (bias) of 0.413 °C. The dashed orange lines indicate the limits of agreement at −0.741 °C and +1.567 °C.

In addition, a linear trend was observed (*p* < 0.001), indicating a higher mean deviation at higher temperatures (i.e., a larger overestimation of temperature) than at lower ones. The Bland–Altman plot (Figure 2) also demonstrated that extreme deviations occurred only in the medium and low temperature range.

**Figure 3:**
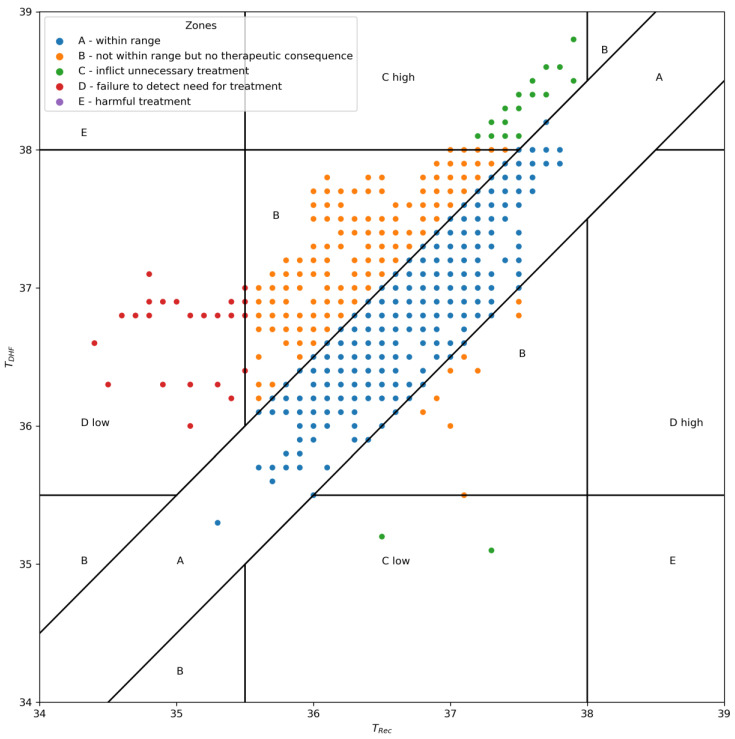
Error grid analysis of DHF temperature (T_DHF_) against rectal temperature (T_Rec_). Zone A represents measurements within an acceptable range, Zone B represents measurements with deviations but without clinical impact, Zone C indicates measurements leading to incorrect clinical decisions, Zone D represents potential failures to detect severe hypothermia or hyperthermia and Zone E represents potential harmful treatment.

Error grid analysis showed that 95.81% of all T_DHF_ measurements did not lead to clinically incorrect decisions and would have led to the same treatment or diagnostic workup. Only 1.70% of the measurements would have led to unnecessary treatment, and 2.49% would have failed to detect the need for treatment (Figure 3).

Throughout the study duration, all patients exhibited good tolerance to the DHF sensor, and no skin reactions were observed.

## 4. Discussion

In this study, 57 patients were enrolled, with a mean age and weight of 26.67 months and 11.53 kg, respectively. The T_DHF_ measurements were, on average, 0.413 °C higher than those of T_Rec_, with broader limits of agreement (−0.741 °C and +1.567 °C) than pre-specified. Higher temperature deviations were observed at higher temperatures. An error grid analysis showed that 95.81% of the T_DHF_ measurements did not result in changes in clinical decisions compared with those of T_Rec_. The T_DHF_ sensor was well-tolerated; no side effects related to the use of the forehead sensor were observed throughout the entire evaluation period.

The management of perioperative core temperatures in pediatric patients is of utmost importance. However, the use of invasive temperature monitoring methods may not always be feasible or appropriate. For instance, in fully anticoagulated patients, nasopharyngeal probes can lead to severe epistaxis [40]; the accuracy of urinary bladder probes decreases with low diuresis [41]; and the smallest urinary temperature catheters are 8 Fg and only appropriate from 4 kg of body weight upward. In such cases, non-invasive alternatives would be desirable to ensure accurate temperature assessment. While non-invasive alternatives, such as DHF temperature probes, have gained attention in recent years, the application of these sensors on pediatric patients, particularly those under the age of 6 years, remains largely unexplored.

Our study findings diverge from those reported in other publications that investigated their use in adults, which showed promising results [28,42]. The temperature measurements obtained from the non-invasive sensors failed to meet the target criteria, which required 95% of the differences to fall within the ±0.5 °C range when compared with rectal readings, in our study population. However, as can be seen in Figure 3, the number of measurements that would have led to wrong clinical management was very low, with 4.19% of the total measurements. Additionally, not all wrong clinical decisions are inherently harmful. For example, new evidence suggests that only hypothermia below 35 °C and not, as previously thought, below 36 °C might negatively affect outcomes [38]. Furthermore, at which point additional warming in hyperthermia becomes harmful is unknown. In any case, none of the measurements would have led to the opposite of the correct treatment, e.g., warming a patient that should have been cooled or vice versa (Zone E). Therefore, from a clinical perspective, the DHF sensor can still be considered for continuous temperature measurement when more invasive methods are unavailable or contraindicated (e.g., in awake children).

Thus far, two studies have examined heat flux thermometers in a pediatric population. First, Evron et al. investigated a mixed population of 16 children and 34 adults and compared the measurements from the Temple Touch Pro™ (TTP) with esophageal measurements [29]. The second study, by Nemeth et al., also compared the TTP measurements to esophageal temperatures in 100 children aged six years or younger [31]. Both studies showed that Temple Touch Pro™ temperatures are sufficiently accurate for routine clinical use. Nemeth et al. showed a mean difference of −0.07 °C (95% CI −0.15 to +0.05), with 95% limits of agreement of −1.00° and +0.858 °C, showing a higher accuracy of the TTP compared to our study. However, there are two noteworthy differences between the previous works and our study. First, their reference probes were placed in the esophagus rather than rectally. Second, their mean patient age was significantly higher, with 34.8 (SD 25.2) months compared with our 26.67 (21.45) months (note that we previously reported the median age in our results due to the skewness of our data). Indeed, the authors of the other studies also reported higher mean differences and a slight tendency to overestimate core temperatures in the neonate subpopulation. Against the background of our younger population, it could be hypothesized that a bias of overestimating the core temperature might be attributable to younger ages.

A recent study of the Temple Touch Pro™ in an adult population showed a tendency to overestimate low core temperatures and underestimate high core temperatures, as well as higher limits of agreement than 0.5 °C, comparable with our findings. In line with our observations, the authors of previous studies have found that DHF measurements are unlikely to lead to wrong clinical decisions and may therefore be a useful alternative for conscious patients undergoing neuraxial or regional anesthesia [43].

In addition to comparing our data with the current literature of DHF sensors, we investigated the relationship of our data with pediatric data from ZHF sensor studies that compared ZHF sensor measurements to esophageal temperatures. Consistently with the studies conducted by Carvalho et al. and Nemeth et al. [31], both on children, we observed a positive bias of +0.41 °C, compared with +0.14 °C and +0.26 °C, respectively. However, we observed wider limits of agreement, of −0.741 °C to +1.567 °C, compared with −0.39 to 0.66 °C and −0.11 to +0.62 °C, respectively. A possible explanation for the higher bias of the DHF to rectal measurements compared to the ZHF/DHF to esophageal measurements is the tendency of rectal temperature probes to underestimate core temperatures as measured with pulmonary artery temperatures compared to esophageal temperature measurements [44]. Additionally, Carvalho et al.—similarly to Nemeth et al.—studied a comparatively older population (mean age of 66.1 months (SD ± 46.8) vs. 26.67 months (SD ± 21.45)). This might have resulted in the same overestimation effect (bias and higher mean difference) discussed previously.

Several explanations for the general overestimation of temperatures with ZHF and DHF sensors in children have been discussed. One hypothesis is that intraoperative warming systems are being positioned closer to the head in young children compared to adults, resulting in a faster warming effect. The correction algorithms of the devices (DHF and ZHF) are based on adult anatomical features, suggesting a possibility of slight overestimation of temperatures in young children, as seen in the study by Carvalho et al., that by Nemeth et al. and our findings [30,45,46].

The relevant limitations of this study need to be addressed. Firstly, it could only assess the accuracy of DHF sensors in representing central core temperatures in pediatric patients by comparing them to a surrogate measure, namely rectal temperature, and not to the gold standard of pulmonary artery catheter temperature. Rectal temperature tends to exceed the core body temperature, primarily due to factors like feces. Moreover, the reliability of rectal measurement is compromised by significant measurement latency when a thermometer is inserted less than 5 cm into adults, as well as during instances of rapid body temperature fluctuation, such as those resulting from substantial volume changes. However, rectal temperature is still considered a well-established standard of care in children for the perioperative setting and is recommended by several guidelines [4,7,19].

Secondly, the limited range of temperature variations and the relatively flat temperature slopes observed in this study hindered the extrapolation of our results to scenarios involving rapid and extreme temperature fluctuations.

A third limitation was the sample size. The significantly higher calculated sample size was neither covered by the vote of the Ethics Committee nor feasible with the allocated budget, leading to a sample size of 51 patients as opposed to the calculated 117. This underenrollment compromised this study’s statistical power, might have made it challenging to detect meaningful effects or differences and could raise concerns about the generalizability of the findings and the increased risk of Type II errors.

On a different note, we look forward to future developments in non-invasive core temperature sensors. For instance, a novel non-invasive heat-flux-based thermometer with wireless functionality holds promise for clinical applications and has already been tested in a clinical setting [47]. Another ZHF-based prototype, designed specifically for warmer environments, may have reduced susceptibility to the influence of nearby convection-based thermal blankets and other warming methods [48].

Further research is thus warranted to determine the efficacy, safety and limitations of DHF temperature probes in this specific population, ultimately facilitating evidence-based temperature management strategies for pediatric patients as well as appropriate sensor calibration.

## 5. Conclusions

In conclusion, the results for the dual-sensor heat-flux (DHF) temperature sensors when used in small children and toddlers undergoing surgery showed a tendency to overestimate core temperatures when compared to rectal temperatures. However, due to the low number of clinically relevant decision errors, this method may still be considered when more invasive methods are contraindicated.

## Figures and Tables

**Table 1:** Study population demographics.

	N = 51 Patients
Age in Months (Quartiles)	
	18.80 (9.37–44.73)

Sex (*n*)	5 females
	46 males
Weight in kg (SD)	
	11.00 (8.50–14.00)

ASA (*n*)	
1	45
2	4
3	2

## Data Availability

Anonymized data will be made available upon reasonable request. Please contact the corresponding author.
